# Korean Red Ginseng Enhances Immunotherapeutic Effects of NK Cells via Eosinophils in Metastatic Liver Cancer Model

**DOI:** 10.3390/nu14010134

**Published:** 2021-12-28

**Authors:** Hee Jung Kwon, Sunyi Lee, Hwan Hee Lee, Hyosun Cho, Joohee Jung

**Affiliations:** 1 Department of Pharmacy, College of Pharmacy, Duksung Women’s University, Seoul 01369, Korea; rnjs973312@duksung.ac.kr (H.J.K.); hyosun1102@duksung.ac.kr (H.C.); 2 Duksung Innovative Drug Center, Duksung Women’s University, Seoul 01369, Korea; sunyi89@duksung.ac.kr (S.L.); leedh01070@duksung.ac.kr (H.H.L.)

**Keywords:** Korean Red Ginseng, eosinophil, metastatic liver cancer, natural killer cell, IL33

## Abstract

Metastasis decreases the survival rate of patients with liver cancer. Therefore, novel anti-metastatic strategies are needed. Korean Red Ginseng (KRG) is often ingested as a functional food with an immune-boosting effect. We investigated a combination of KRG and natural killer (NK) cells as a novel immunotherapy approach. SK-Hep1 cells were injected into the tail vein of NRGA mice to establish an experimental metastasis model. KRG, NK cells, or a combination of KRG and NK cells were administered. Tumor growth was observed using an in vivo imaging system, and metastatic lesions were evaluated by histological analysis and immunohistochemistry. Bioluminescence intensity was lower in the KRG and NK cell combination group than in the other groups, indicating that the combination treatment suppressed the progression of metastasis. CD56 expression was used as a NK cell marker and hematological analysis was performed. The combination treatment also decreased the expression of matrix metalloproteinases and the area of metastatic lesions in liver and bone tissues, as well as increased the eosinophil count. Expression of cytokines-related eosinophils and NK cells was determined by Western blotting analysis. The expression of interleukin 33 (IL33) was induced by the combination of KRG and NK cells. High IL33 expression was associated with prolonged overall survival in the Kaplan–Meier plotter. Our results suggest that KRG enhances the immune activity of NK cells by IL-33 through eosinophils and suppresses metastatic liver cancer progression.

## 1. Introduction

Liver cancer is a major cause of death worldwide. Risk factors for liver cancer include chronic hepatitis B or C infection [1]. Primary liver cancer proliferates and progresses through infiltration and invasion. Metastasis occurs as invading liver cancer cells circulate in blood vessels, extravasate, and form colonies at secondary sites [2], mainly in the lungs, portal veins, bones, and portal lymph nodes [3]. Metastatic liver cancer has a poor prognosis and anti-metastatic strategies are needed to improve the overall survival (OS) of patients with liver cancer.

Chemotherapy, targeted therapy, and immunotherapy are used to treat metastatic liver cancer. Immunotherapy activates the immune system, which then targets and kills cancer cells [4] via the T cells, B cells, macrophages, and natural killer (NK) cells present in the tumor microenvironment. However, the function or expression of these immune cells is often altered by cancer cells [5,6]. Therefore, immunotherapy is only effective in limited cancer patients. To increase the effectiveness of immunotherapy, activated immune cells are required and the immune system should be modulated. For instance, activated NK cells enhance the anti-cancer effects of T cells and induce cytotoxicity in liver cancer cells [7]. 

Functional foods or adjuvant drugs have been investigated to boost the activity of NK cells and improve immunotherapeutic efficacy [8]. Korean Red Ginseng (KRG) is known as an immune-enhancing agent [9,10] with antioxidant activity [11]. No observed adverse effect level (NOAEL) is reported to be over 2000 mg/kg in mice [12]. Thus, KRG is often ingested as a functional food. In this study, we investigated whether KRG enhanced immunotherapy of NK cells in the metastatic liver cancer xenograft model. 

## 2. Materials and Methods

### 2.1. Animals

All animal experiments were approved by the Institutional Animal Care and Use Committee of Duksung Women’s University (2019-003-009) in accordance with the guidelines for the care and use of laboratory animals. Five-week-old male NOD/ShiLtJ-Rag2^em1AMC^Il2rg^em1AMC^ (NRGA) mice were purchased from JA BIO (Suwon, Korea). Mice were acclimatized for 1 week before experiments. Laboratory conditions were maintained at 20 °C with 50% humidity and a 12 h light/12 h dark cycle. Mice were provided drinking water ad libitum.

### 2.2. Establishment of Experimental Metastatic Liver Cancer Model

SK-Hep1_Luc cells (human liver cancer cells expressing luciferase) provided by Prof. S. Kuroda (Osaka University, Japan) were maintained in Dulbecco’s modified Eagle’s medium (Thermo Fisher, Waltham, MA, USA) supplemented with 10% fetal bovine serum (FBS, GW Vitek, Seoul, Korea) and 1% penicillin-streptomycin (GenDEPOT, Katy, TX, USA). All cells were cultured in a 5% CO_2_ incubator at 37 °C. SK-Hep1_Luc cells (1 × 10^6^ cells/100 μL) were intravenously injected into the tail vein of NRGA mice as slowly as possible. 

### 2.3. Treatment

KRG extract was used as CheongKwanJang (Korea Ginseng Corporation, Daejeon, Korea). KRG extract was made by decocting KRG roots cultivated over six years and contained ginsenoside Rg1, Rb1, and Rg3 (5 mg/g). KRG extract (50 mg/kg BW/10 mL) or water (control group) was orally administered daily after transplantation of SK-Hep1_Luc cells.

NK-92 cells (human NK cells, ATCC, CRL-2407) were maintained in alpha minimum essential medium (Gibco, Waltham, MA, USA) supplemented with 20% FBS (GW Vitek), 1% penicillin-streptomycin (GenDEPOT), 0.1 mM 2-mercaptoethanol (Sigma-Aldrich, Inc., St. Louis, MO, USA), and 100–200 U/mL rIL-2 (Biolegend, San Diego, CA, USA). NK cells (2 × 10^6^ cells/100 μL) or phosphate-buffered saline (PBS; control group) were intraperitoneally injected twice a week beginning on day 20.

### 2.4. In Vivo Imaging

To monitor metastasis, bioluminescence imaging of luciferase activity was conducted using an in vivo system (Visque^®^ InVivo Elite, Vieworks, Gyeonggi, Korea). Mice anesthetized with 2% isoflurane (Piramal Pharma Solutions, Mumbai, India) were intraperitoneally injected with D-luciferin (150 mg/kg, PerkinElmer, Waltham, MA, USA). After 15 min, photos were taken twice a week with an exposure time of 10 min. These evaluation experiments in the metastasis model were repeated twice. 

### 2.5. Hematoxylin and Eosin Staining

Tissues from metastatic lesions in the liver and epiphysis were isolated and fixed in 4% para-formalin solution. Bones were demineralized. Paraffin-embedded liver or bone blocks were cut into 3 μm thick sections, which were stained with hematoxylin and eosin (H&E) solution (Sigma-Aldrich) and observed via microscopy (Leica Microsystems, Ltd., Wetzlar, Germany).

### 2.6. Immunohistochemistry

Tissue sections were treated with a 0.3% H_2_O_2_ solution for 10 min and rinsed in PBS. Next, heat-induced antigen retrieval was performed by incubating the sections for 25 min in target retrieval buffer (pH 9.0) at 95 °C. The sections were stained with anti-NCAM1 (CD56) antibody (1:100; Cell signaling technology, MA, USA), anti-matrix metalloproteinase (MMP)2 antibody (1:200; Millipore, Burlington, MA, USA), anti-MMP7 antibody (1:200; Santa Cruz Biotechnology, Dallas, TX, USA), anti-MMP9 antibody (1:200; Abcam, Cambridge, UK), and anti-Ki67 antibody (1:200, Abcam) overnight at 4 °C. Antigen-antibody reactions were visualized using EnVision+ Dual Link System-HRP (Vector Laboratories, CA, USA) and 3,3′-diaminobenzidine (Vector Laboratories). The stained sections were dehydrated, counterstained with hematoxylin, and mounted in limonene mounting medium (Abcam).

### 2.7. Hematological Analysis

On day 30, mice were anesthetized with 2% isoflurane. Blood obtained from the aorta of mice was collected in EDTA-containing tubes. Hematological tests were performed using an automated hematology analyzer (ADVIA 2120i, Siemens Healthcare Diagnostics, Munich, Germany).

### 2.8. Western Blotting

Liver tissues were lysed in RIPA buffer (GenDEPOT) with protease inhibitors (P3100, GenDEPOT) and phosphatase inhibitors (P3200, GenDEPOT). Proteins (100 μg) were separated by 12% SDS-PAGE at 120 V for 150 min using Mini-Protean Tetra cell and PowerPac^TM^ Basic Power supply (BioRad, Hercules, CA, USA) and transferred to a polyvinylidene difluoride (PVDF) membrane (Millipore, Darmstadt, Germany) at 0.15 A for 90 min using Novex^®^ semi-dry blotter (Invitrogen Co., Carlsbad, CA, USA). The membranes blocked with 5% skimmed milk in Tris-buffer including 0.1% Tween 20 were incubated with primary antibodies against interleukin 33 (IL33, R&D system, Minneapolis, MN, USA, 1:1000), major basic protein (MBP, MyBioSource, San Diego, CA, USA, 1:1000), interferon γ (IFNγ, Santa Cruz Biotechnology, 1:1000), and β-actin (Sigma-Aldrich, 1:5000) overnight at 4 °C, and incubated with secondary anti-mouse antibodies (1:3000) for 3 h at room temperature. Protein was visualized using the enhanced chemiluminescent solution and detected by a Chemi-doc (FluorChem E system, San Jose, CA, USA).

### 2.9. Measurement of CD56^dim+^ NK Cells

All antibodies were purchased from BD biosciences (Franklin Lakes, NJ, USA). Briefly, the blood of mice was harvested to assess the density of NK-92 cells within the blood and separated into lymphocytes. Then, the lymphocytes (1 × 10^6^ cells) were stained with anti-human CD56-APC and anti-VP-PerCP (1:4) for 30 min. After staining, the cells were analyzed using flow cytometry (Novocyte^®^ Flow Cytometer, ACEA Biosciences, San Diego, CA, USA). The population of CD56^dim+^ NK-92 cells was compensated by a comparison of each emissive value.

### 2.10. Kaplan–Meier Plotter Database Analysis

OS and IL33 expression data from liver cancer patients (ID: 90865, 364 patients) were obtained from the Kaplan–Meier plotter database (http://kmplet.com/analysis/, accessed on 10 December 2021). The data included all stages, grades, gender, and race and were analyzed by the best cutoff. Hazard ratios (HRs) with 95% confidence intervals and log-rank *p*-values were calculated using the Kaplan–Meier plotter.

### 2.11. Statistics

Data are expressed as the mean ± standard deviation (SD). The data were analyzed via ANOVA using Prism7 (GraphPad Software Inc., San Diego, CA, USA). Values of *p* < 0.05 were considered statistically significant.

## 3. Results

### 3.1. Combination of KRG and NK Cells Suppressed Metastatic Liver Cancer Progression

**Figure 1:**
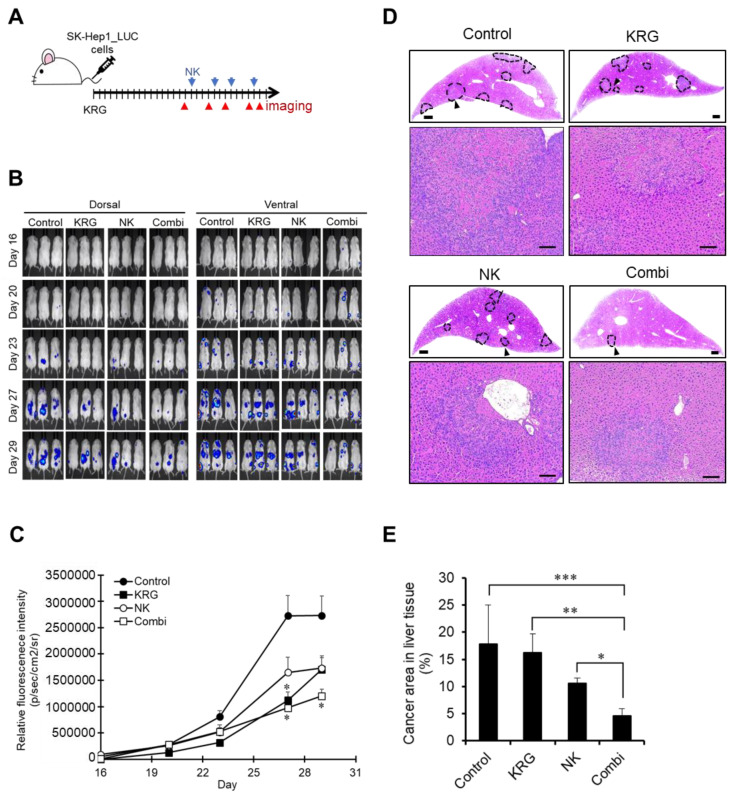
Anti-metastatic effect of KRG and NK cells. (**A**). Schedule of treatment and in vivo imaging in metastasis model (*n* = 3/group). (**B**). In vivo imaging of SK-Hep1 metastasis. The in vivo bioluminescence imaging was conducted on days 16, 20, 23, 27, and 29. Images are representative of twice replicated experiments. (**C**). In vivo bioluminescence intensity. Data obtained from B are expressed as the mean ± SD (*n* = 3/group). *, *p* < 0.05 (one-way ANOVA with a Dunnett’s post hoc test). (**D**). Hematoxylin staining of liver tissues. Dashed lines indicate metastatic lesions. Scale bar, 500 μm (Upper panel). Magnified pictures of the arrow point are shown in the lower panel. Scale bar, 100 nm. (**E**). Area of metastatic lesions in liver tissues. Data are expressed as the mean ± SD (two slides of liver tissue from each mouse, *n* = 3/group). *, *p* < 0.05; **, *p* < 0.01; ***, *p* < 0.005. (two-way ANOVA with a Tukey’s post hoc test).

Each group was randomly divided, and then SK-Hep1_Luc cells were intravenously injected. Every day, KRG was orally treated. On day 16, SK-Hep1 cell-derived metastasis was just observed. NK cells were intraperitoneally injected the next day (Figure 1A). Metastasis progression was evaluated by analyzing luciferase activity in SK-Hep1_Luc cells. As shown in Figure 1B, bioluminescence was observed in bone (mainly the spine and femur) and liver tissues. On and after day 20, metastatic liver cancer was observed and increased in the control group. On days 27 and 29, metastasis progression was delayed in all three treatment groups. In particular, KRG and the combination of KRG and NK cells significantly inhibited metastasis (*p* < 0.05) compared to control on day 27, and the combination of KRG and NK cells significantly maintained the suppression of metastasis on day 29 (Figure 1C). 

All three treatment groups showed the reduction of metastatic lesions in liver tissues (Figure 1D). Unfortunately, KRG or NK cells-treated groups showed no significant reduction of metastatic area, whereas the combination group with KRG and NK cells exhibited a statistically significant reduction in lesion area of liver tissues (Figure 1E).

### 3.2. Combination of KRG and NK Cells Inhibited the Expression of Ki67 and MMPs in Metastatic Lesions

**Figure 2:**
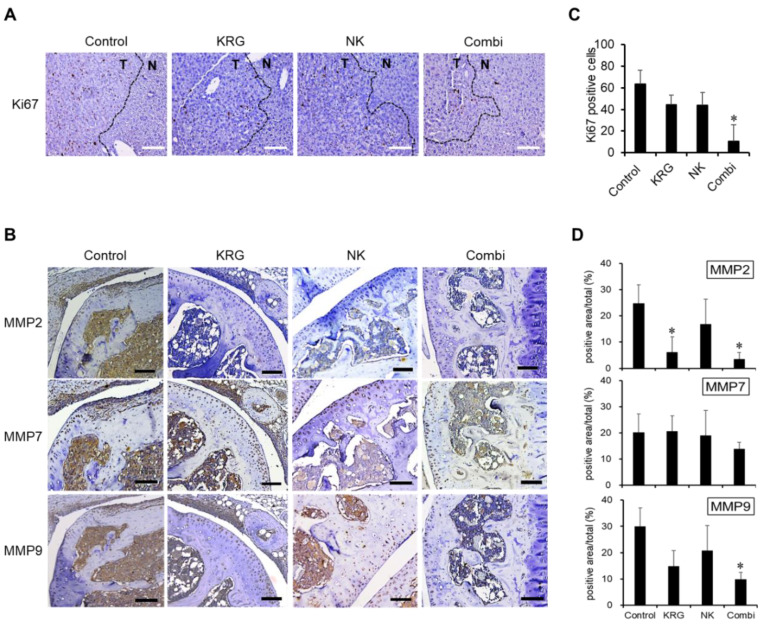
Inhibition of matrix metalloproteinases and Ki67 levels by KRG and NK cells. (**A**). Expression of Ki67 in metastatic lesions of liver tissues. Brown dots represent Ki67-expressing cells. T, tumor site; N, normal site. Scale bar, 100 μm. (**B**). Quantification of Ki67-expressing cells in the tumor site. Data are expressed as the mean ± SD (randomly three points in each slide. *n* = 2/group). *, *p* < 0.05 (one-way ANOVA with a Dunnett’s post hoc test). (**C**). Expression of matrix metalloproteinases (MMPs) in metastatic lesions of the epiphysis. Brown dots represent MMP-expressing cells. Scale bar, 100 μm. (**D**). Quantification of MMPs in the metastatic lesions. Data are expressed as the mean ± SD (randomly three points in each slide. *n* = 2/group). *, *p* < 0.05 (*vs* control, two-way ANOVA with a Tukey’s post hoc test).

To confirm the effect on metastatic lesions and evaluate anti-metastatic efficacy, sections of the liver and femur were analyzed. The proliferative and invasive activity was determined by measuring the expression of Ki67 and MMPs. Ki67 expression was observed only in metastatic lesions in liver tissues (Figure 2A, T region) and was significantly decreased by KRG, NK cells, and the combination treatment (Figure 2A,B). The combination treatment of KRG and NK cells showed a synergic effect (Figure 2B).

MMP2, MMP7, and MMP9 expression were observed in the epiphysis (Figure 2C). MMP2 expression was inhibited by KRG, NK cells, and the combination treatment, but MMP7 expression showed no change. MMP9 expression was decreased by KRG and the combination treatment. The levels of MMP2 and MMP9 expression in bone were correlated with that of Ki67 in liver tissues. The combination of KRG and NK cells suppressed Ki67, MMP2, and MMP9 expression levels more than KRG or NK cells alone (Figure 2B,D). These results suggest that the combination of KRG and NK cells inhibited the growth of SK-Hep1–derived tumors and produced anti-metastatic effects.

### 3.3. KRG Enhanced Anti-Metastatic Effects of NK Cells through Eosinophils

**Figure 3:**
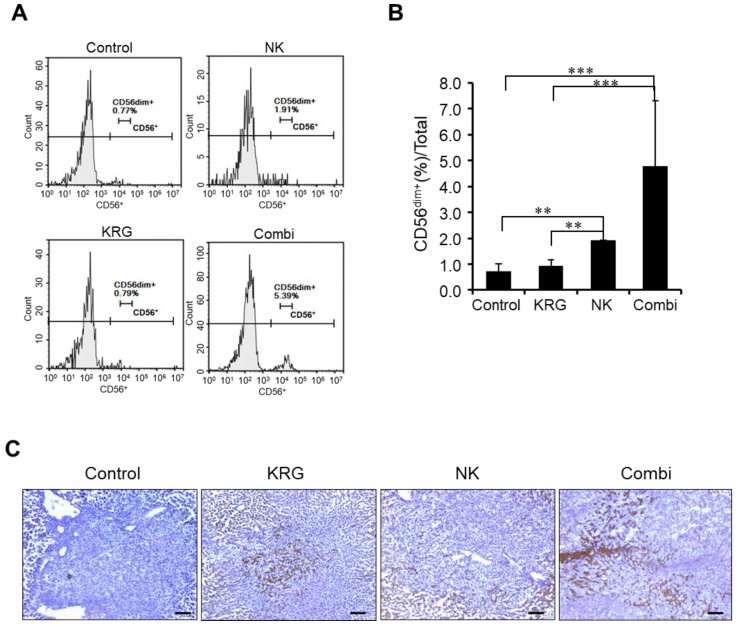
Detection of circulating NK cells in an experimental metastasis model. (**A**). CD56 levels measured via FACS analysis. (**B**). Ratio of CD56^dim+^ cells. Data are expressed as the mean ± standard deviation (*n* = 3/group). **, *p* < 0.01; ***, *p* < 0.005. (two-way ANOVA with Tukey’s post hoc test) (**C**). CD56 positive cells in liver tissues. Brown dots represent CD56 positive cells. Scale bar, 100 μm.

In the metastatic liver cancer model, the combination of KRG and NK cells was determined to be effective immunotherapy. Therefore, we performed hematological analysis to investigate the mechanism of action of KRG and NK cells in the blood. CD56 ^dim+^ expression was used as a positive biomarker for circulating NK cells and was measured by FACS analysis (Figure 3A). CD16 expression was not detected because NK-92 cells are known to be negative. As shown in Figure 3B, groups treated with NK cells and the combination treatment exhibited significantly increased CD56 ^dim+^ expression, indicating that intraperitoneally injected NK cells remained in the blood. In liver tissues, CD56 positive cells including NK cells were investigated (Figure 3C). The control group was CD56 negative in the liver tissues including the tumor, but the combination of KRG and NK cells showed penetrated CD56 positive cells in the tumor tissue. In particular, the NK cells-only-treated group showed CD56 positive cells around the tumor tissue, not in the tumor. KRG only-treated group slightly showed CD56 positive cells in the tumor tissue; on the other hand, the combination group increased CD56 positive cells in the tumor tissue. These results suggested that KRG enhanced the penetration of CD56 positive cells as NK cells into the tumor tissue.

**Figure 4:**
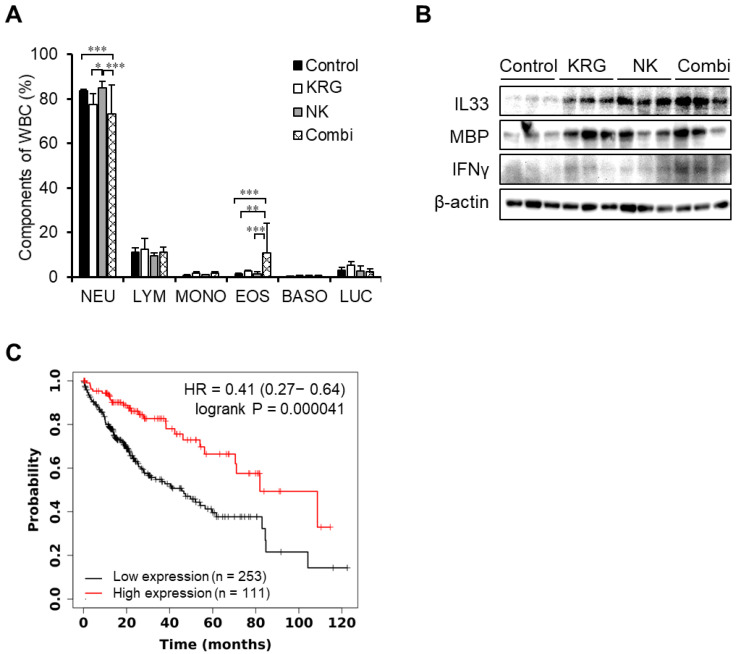
Activation of IL33 related to eosinophils in an experimental metastasis model. (**A**). Ratio of white blood cell (WBC) components. Data are expressed as the mean ± standard deviation (*n* = 3/group). *, *p* < 0.05; **, *p* < 0.01; ***, *p* < 0.005. (two-way ANOVA with Tukey’s post hoc test) (**B**). Expression of IL33, MBP, and IFNγ levels in metastatic liver tissues. (**C**). Kaplan–Meier curve of liver cancer patients with high (*n* = 111) and low (*n* = 253) IL33 expression.

The combination of KRG and NK cells also caused a change in blood composition (Table 1). NK cell treatment produced no change in blood composition, while KRG treatment decreased the number of platelets. Conversely, the combination of KRG and NK cells increased the number of platelets. On the other hand, the white blood cell (WBC) count increased in KRG treatment, and decreased in the combination of KRG and NK cells, although these results showed no significance. In the combination of KRG and NK cells, the percentages of neutrophils and eosinophils were significantly decreased and increased, respectively (Figure 4A). These results suggest that KRG regulates hematological parameters in tandem with NK cells.

To investigate whether KRG affects the activities of eosinophils and neutrophils, the expression of cytokines, IL33, MBP, and IFNγ was measured. As shown in Figure 4B, the combination treatment increased IL33, MBP, and IFNγ expression levels, suggesting that an increased ratio of eosinophils and high IL33 expression confers an anti-metastatic effect. To investigate whether IL33, MBP, and IFNγ are associated with the OS of liver cancer patients, the correlation between the expression of these genes and OS was analyzed using the Kaplan–Meier plotter. High IL33 expression was associated with extended OS (Figure 4C, HR = 0.41, *p* = 0.000041), indicating a positive correlation between IL33 expression and OS in liver cancer patients. However, MBP and IFNγ expression showed no significance in OS of liver cancer patients (data not shown).

## 4. Discussion

In the SK-Hep1–derived metastatic liver cancer model, the combination of KRG and NK cells significantly delayed metastasis, while treatment with KRG or NK cells alone exhibited only a slight anti-metastatic effect. By monitoring the bioluminescence level in vivo, anti-metastatic effects were observed, including decreased Ki-67 expression and reduced metastatic lesion area in liver tissues. 

To prevent metastasis, the infiltration and invasion of primary cancer cells should be inhibited. We examined the expression of MMPs, which are important factors of tumor invasion. MMP2 and MMP9 are predictors of liver cancer recurrence [13] and are highly expressed in invasive hepatocellular cancer (HCC) cells [14]. Inhibition of MMP2 and MMP9 produces anti-metastatic effects in breast cancer [15] and HCC [16]. In our study, MMP2 and MMP9 expression levels were decreased by treatment with KRG and the combination of KRG and NK cells. Consistent with our results, red ginseng extract has been shown to inhibit MMP2 and MMP9 expression in colon cancer cells [17] and adipocytes [18]. Therefore, our results indicate that KRG inhibited metastasis by decreasing MMP2 and MMP9 expression, and the combination of KRG and NK cells enhanced the anti-metastatic effect. On the other hand, NK cells are known to induce the expression of MMP2 and MMP9 [19]. Our results also showed that in the group treated with NK cells, the expression of MMP2, MMP7, and MMP9 was similar to that observed in the control group. Nevertheless, the combination of KRG and NK cells produced greater anti-metastatic effects than those conferred by treatment with KRG or NK cells alone. 

We further studied the relationship between KRG and NK cells. NK-92 cells used in this study are known to express CD2 and CD56, but not CD3, CD4, CD8, and CD16 [20]. Thus, the presence of NK cells was confirmed by detecting only CD56 expression in severe immune-deficient mice. The circulation of injected NK cells could be observed in blood while monitoring the in vivo system. The combination of KRG and NK cells increased the number of CD56^dim+^ cells. Furthermore, the combination of KRG and NK cells increased the penetration of CD56 positive cells into the tumor tissue. To elucidate improving the anticancer effect of NK cells by KRG in the metastatic liver cancer model, immune cell networking was investigated. In the presence of NK cells, the numbers of platelets, neutrophils, and eosinophils showed no change compared to the control group. The combination of KRG and NK cells induced changes in these cells. Platelets are known to disrupt NK cell function [21], aid the circulation of malignant cells [22], and promote cancer progression [23]. KRG is known to inhibit platelet activation [24] and prevent cardiovascular diseases [25]. Our results also showed that the number of platelets was decreased in the group treated with KRG. However, the platelet count was increased in the group treated with the combination of KRG and NK cells. The mechanism by which the combination of KRG and NK cells increased the number of platelets is unclear. The decrease in the number of platelets could have occurred as a result of neoplasia [26]. Thus, the results might be that the combination of KRG and NK cells restores the immune system. The platelet number observed after treatment with the combination of KRG and NK cells was within the normal range of healthy mice. 

Among WBCs, the ratio of neutrophils and eosinophils was significantly altered by the combination of KRG and NK cells; the number of eosinophils was increased although total WBC was decreased (no significance). The role of eosinophils in cancer is controversial [27]. Eosinophils infiltrate cancer cells and secrete various angiogenic or cytotoxic molecules, depending on the state of the tumor microenvironment and the tumor type [28,29]. In colorectal cancer [30] and HCC [31], eosinophils exhibit anti-tumorigenic activities. Granule proteins of eosinophils cause cytotoxicity [32]. Among them, MBP activates mast cells, basophils, and neutrophils during the process of inflammation [33] and acts as a platelet agonist [34]. Our results indicate that the MBP level is increased by the combination of KRG and NK cells. 

Eosinophils are activated by IL33, IFN-γ, and IL-5 [28], and activated eosinophils secrete MBP, tumor necrosis factor (TNF)-α, eosinophil peroxidase, and granzyme B, which facilitate the killing of tumor cells [35]. The mechanism of the effect of IL33 on tumor progression is still controversial [36]. In breast cancer [37], cholangiocarcinoma [38], and colon cancer [39], IL33 promotes cancer progression. IL33 has also been shown to promote the infiltration of CD8+ T cells into tumor cells and suppress tumor growth in a melanoma model [40]. IL33 also activates NK cells and inhibits metastasis [41]. We found that eosinophils activated by IL33 secreted MBP, indicating that the combination of KRG and NK cells suppressed metastatic liver cancer cells. We also observed that the combination of KRG and NK cells decreased the number of neutrophils, although KRG was previously reported to cause an accumulation of neutrophils [42]. KRG is known to activate NK cells in non-alcoholic fatty liver disease [43]. Our results suggest that KRG also activates NK cells through IL33 and that activated NK cells increase IFNγ in the metastatic liver cancer model. 

The experimental metastasis is established by intravenous injection of cancer cells in NRGA mice which are total immune-deficient (absent B cells, T cells, and NK cells) mice. Thus, the transplantation of human-derived cancer cells is available in NRGA mice. This study could evaluate the effect of human NK cells injected in the metastatic model. Our results might be limited as the experimental metastasis model could not fully recapitulate tumor environment and metastatic liver cancer progression. Nevertheless, these results could provide basic evidence for application in clinical immunotherapy.

## 5. Conclusions

KRG inhibited metastasis and enhanced the anti-metastatic effect of NK cells in the metastatic liver cancer model. The anti-metastatic effect of the combination of KRG and NK cells occurred via IL33 activation, which increased the eosinophil count and secretion of MBP and IFNγ. In particular, a high IL33 level was associated with prolonged OS among liver cancer patients. These results suggest that KRG enhances the immunotherapeutic effects of NK cells in the metastatic liver cancer model.

## Figures and Tables

**Table 1:** Hematological analysis.

	Group	Control	KRG	NK Cells	Combination
Parameter					
RBC (×10^6^ cells/μL)		8.58 ± 0.66	8.06 ± 0.3	7.41 ± 1.06	7.95 ± 0.15
HGB (g/dL)		14 ± 0.9	13 ± 0.36	12.87 ± 0.51	10 ± 5.74
HCT (%)		47.3 ± 4.29	44.43 ± 0.45	40.6 ± 6.52	42.78 ± 1.79
RDW (%)		15.27 ± 0.75	15.4 ± 0.62	15.33 ± 0.55	14.63 ± 0.62
MPV (fL)		8.5 ± 1.67	12.57 ± 3.19	11.03 ± 3.32	8.2 ± 1.36
PLT (×10^3^ cells/μL)		257 ± 51.26	158.67 ± 27.54 *	213.67 ± 136.7	484.5 ± 112.2 *
WBC (×10^3^ cells/μL)	NEU	1.24 ± 0.18	1.33 ± 0.17	1.23 ± 0.24	0.93 ± 0.4
	LYM	0.16 ± 0.03	0.23 ± 0.11	0.13 ± 0	0.14 ± 0.06
	MONO	0.01 ± 0.05	0.03 ± 0.02	0.02 ± 0.01	0.02 ± 0.01
	EOS	0.02 ± 0.01	0.05 ± 0.01	0.03 ± 0.02	0.10 ± 0.09
	BASO	0.0	0.01 ± 0.01	0 ± 0.01	0.01 ± 0.01
	LUC	0.04 ± 0.02	0.09 ± 0.03	0.04 ± 0.02	0.03 ± 0.02
	Total	1.48 ± 0.21	1.73 ± 0.29	1.44 ± 0.24	1.23 ± 0.43

RBC, red blood cell; HGB, hemoglobin; HCT, hematocrit; RDW, red cell distribution width; MPV, mean platelet volume; PLT, platelet; WBC, white blood cell; NEU, neutrophil; LYM, lymphocyte; MONO, monocyte; EOS, eosinophil; BASO, basophil; LUC, large unstained cell. *, *p* < 0.05 (one-way ANOVA with a Dunnett’s post hoc test).

## Data Availability

The dataset supporting the conclusions of this article is included within the article.
